# “Common questions and misconceptions about dietary supplements and the industry - What does science and the law really say?“

**DOI:** 10.1080/15502783.2025.2534128

**Published:** 2025-07-15

**Authors:** Jose Antonio, Brandi Antonio, Alan Aragon, Erik Bustillo, Darren Candow, Rick Collins, Edwin Davila, Bob Durkin, Douglas Kalman, Chris Lockwood, Scotty Mills, Jeffrey R. Stout

**Affiliations:** a NSU Florida, Department of Health and Human Performance, College of Health Care Sciences, Davie, FL, USA; b University of Central Florida, School of Kinesiology and Rehabilitation Sciences, Orlando, Florida, USA; c LLC. Chatsworth, Fit Advancement, California, USA; d Concordia University Chicago, Department of Exercise Science, Fit Advancement, River Forest, Illinois, USA; e University of Regina, Faculty of Kinesiology & Health Studies, Regina SK, Canada; f Collins Gann McCloskey & Barry PLLC. Mineola, New York, USA; g Precision Health, 7600 Broadway Unit 5023, San Antonio, Texas, USA; h Amin Wasserman Gurnani, Precision Health, Washington DC, USA; i NSU Florida, Department of Nutrition, College of Osteopathic Medicine, Davie, Florida, USA; j Woodbolt Distribution LLC dba Nutrabolt, Department of Nutrition, College of Osteopathic Medicine, Austin, Texas, USA

**Keywords:** Social media, regulation, FDA, safety, nutrition

## Abstract

Dietary supplement use is popular among fitness enthusiasts as well as competitive athletes. There is, however, confusion regarding the regulatory framework as well as the basic science regarding the use of supplements. Although there is an extensive body of scientific and legal writings on dietary supplements, several misconceptions persist vis-à-vis this category. Thus, the following questions will be addressed in this review. 1) Are dietary supplements regulated by the Food and Drug Administration? 2) Are foods and supplements regulated similarly? 3) What is the role of the Federal Trade Commission? 4) Besides federal regulations for dietary supplements, do state laws also regulate the category? 5) If a supplement company funds a study, does that automatically call into question the results? 6) Can diet alone provide everything you need without using supplements? 7) Is it necessary to conduct randomized controlled trials (RCTs) on dietary supplements? 8) How safe are supplements compared to OTC drugs? 9) Where can consumers find accurate information about supplements? 10) Why does the NIH fund dietary supplement research related to disease, yet findings cannot be marketed by supplement companies? 11) What is the size of the dietary supplement industry compared to the pharmaceutical industry? 12) How can I know if a dietary supplement is safe and free of banned substances? Similar to our prior papers, a team of legal and science scholars evaluated the evidence on these salient questions.

## 1. Introduction

The use of dietary supplements is quite common in the world of athletics. For instance, among all sports in the Olympic Games (Atlanta and Sydney), boxing had the highest prevalence of vitamin use in Atlanta (91%), whereas swimming led in Sydney (76%). The highest use of mineral supplements was observed among rowers (56%) and cyclists (73%). Nutritional supplements were most frequently used in swimming (56%) and reached 100% among cyclists [1]. More recently, an observational study of 1,392 athletes found that 70% of Olympic athletes and 50% of Winter Paralympians reported using supplements from the Tokyo 2020 and Beijing 2022 Olympic and Paralympic Games [2].

Nonetheless, dietary supplement use is somewhat controversial, and their regulation, safety, and efficacy remain subjects of widespread debate. Governed in the U.S. by the Dietary Supplement Health and Education Act (DSHEA) of 1994, supplements are regulated as a subset of foods by the Food and Drug Administration (FDA), with additional oversight from the FTC regarding advertising claims. Unlike pharmaceuticals, dietary supplements do not require pre-market approval, but manufacturers must ensure safety and comply with labeling standards, Good Manufacturing Practices (GMPs), and adverse event reporting. Critics argue this regulatory framework prioritizes post-market enforcement, creating gaps in consumer protection, while proponents highlight its flexibility in fostering innovation. As the $54 billion supplement industry grows, its interplay with pharmaceuticals ($670 billion) and evolving regulations highlights both opportunities and challenges. Therefore, the purpose of this review is to elucidate the scientific and regulatory framework regarding the use of dietary supplements and to address several salient questions regarding this category. Furthermore, it should be noted that the regulatory framework in which we address several of these questions is based in the United States and may not apply to other nations or jurisdictions.

### 1.1. Are dietary supplements regulated by the food and drug administration?

Although some media sources may suggest otherwise, dietary ingredients and dietary supplements are regulated by the Food and Drug Administration (FDA) as unique types of food substances and food, respectively. Although each of these terms has been used in common parlance for some time, a formal regulatory definition under the Federal Food, Drug, and Cosmetic Act (FDCA) [3], Pub. L. No. 75–717, 52 Stat. 1040 (1938) did not exist until 15 October 1994—the effective date of the Dietary Supplement Health and Education Act (DSHEA), Pub. L. No. 103–417, 108 Stat. 4325 (1994) [4]. Prior to DSHEA, the FDA tended to regulate dietary ingredients as food additives and too often viewed the dietary supplements that contained them as adulterated food for containing an unsafe/unapproved food additive. DSHEA, which was unanimously passed by Congress, brought unique statutory and regulatory requirements for dietary ingredients and the dietary supplements that contain them.

Under DSHEA, a dietary supplement is legally defined as a product that may include vitamins, minerals, herbs or other botanicals, amino acids, and other substances such as enzymes, organ tissues, glandulars, or metabolites [5]. Supplements are intended for oral consumption (i.e. via forms such as tablets, capsules, powders, softgels, or liquids) and must be labeled as a “dietary supplement” and not represented as a conventional food [5]. It should be noted that supplements are not required to have FDA approval prior to marketing unless they contain a new dietary ingredient (NDI).

For example, DSHEA introduced the definition of a dietary supplement in section 201(ff) of the Act), 21 U.S.C. § 321(ff). Among other things, this definition requires that a dietary supplement contain at least one dietary ingredient, be swallowed into the alimentary canal, not mimic or be intended to take the place of a conventional food, and not contain a dietary ingredient that was approved as a drug or studied as a drug prior to being marketed as a dietary ingredient.

DSHEA also amended the FDCA to add specific sections defining when a product labeled as a dietary supplement is either adulterated or misbranded. Under section 402(f) of the Act, 21 U.S.C. § 342(f), a dietary supplement is adulterated if it presents an unreasonable risk of illness or injury according to the conditions of use on its label. A dietary ingredient that was not marketed in the U.S. as a dietary ingredient or dietary supplement on 15 October 1994, is considered a “New Dietary Ingredient,” or “NDI,” 21 U.S.C. § 350b(d) [6]. A dietary supplement containing a New Dietary Ingredient (NDI) is considered adulterated if it is marketed before meeting the requirements of section 413(a) of the Act (21 U.S.C. § 350b(a)). Specifically, this means that either the NDI must already be present in the food supply in a form that has not been chemically altered, or a New Dietary Ingredient Notification (NDIN) for the NDI or the product containing it must have been submitted to the FDA. The FDA also regulates other aspects of the supplement industry. For example, products must be marketed in accordance with current Good Manufacturing Practices, and the FDA conducts inspections of industry facilities to audit compliance, 21 U.S.C. § 342(g), 21 U.S.C. § 374. Supplement products must also be labeled in compliance with FDA regulations, including the nomenclature and order of ingredients, the placement of information, and even down to the relative font size of words, 21 C.F.R. Part 101. Further, marketing claims and materials must comply with both FDA and FTC regulations.

As with all products regulated under the FDCA, the FDA can take enforcement actions to remove from the market dietary supplements that are adulterated or misbranded and present a risk to public health. These options include the issuance of Warning Letters, seizure of products, and court-ordered injunctions intended to prevent repeated bad behavior. For example, the FDA issued a final rule in 2004 declaring dietary supplements containing ephedrine alkaloids adulterated because they presented an unreasonable risk of illness or injury. That same year, the FDA announced that dietary supplement products containing androstenedione were adulterated new dietary ingredients and sent Warning Letters to companies making, marketing, or selling the products. The FDA can also bring criminal charges against bad actors in the industry through the Department of Justice. While some critics suggest that the FDA lacks the legal authority to properly police the market, there is ample authority for the FDA to do so, and any deficits are primarily due to a lack of enforcement of the existing law.

***In summary, the primary regulatory body responsible for the dietary supplement industry is the FDA (as well as the FTC). Key aspects of FDA regulation include, but are not limited to, new dietary ingredients, labeling requirements, and safety monitoring.***

### 1.2. Are foods and supplements regulated similarly?

Yes, the FDA’s regulation of foods and supplements can be very similar. However, DSHEA provided “certain exceptions” pertaining to the regulation of dietary supplements relative to conventional foods that can have a profound impact, including, but not limited to, the manner in which a product is concluded to be safe, the way it is manufactured, what is required or perhaps not allowed to be on its label, what claims can be made for it, and more.

Some brief examples include:

- Basis for safety: The basis of safety required to use an ingredient as a food additive or GRAS (“Generally Regarded As Safe”) substance is that it be “reasonably expected to cause no harm” according to its intended use in food. The basis of safety required for a dietary ingredient, or a dietary supplement is that it is “reasonably expected to be safe” according to the conditions of use on the product’s label.
- Adverse event monitoring: The manufacturer, packer, or distributor whose name and address appear on the label of a dietary supplement marketed in the United States is required to submit to the FDA within 14 days all serious adverse events reported to the company as being associated with the use of the dietary supplement in the United States. It must maintain records of all non-serious adverse events reported to it and make these records available to the FDA upon request. Manufacturers of conventional foods must report to the FDA’s Reportable Food Registry, an electronic portal for industry, when there is a reasonable probability that an article of food will cause serious adverse health consequences.
- Manufacturing: Food additives, GRAS substances, and dietary ingredients must be manufactured according to the requirements of 21 C.F.R. Part 117, “Current Good Manufacturing Practice, Hazard Analysis, and Risk-Based Preventive Controls for Human Food.” Dietary supplements, however, must be manufactured according to the requirements of 21 C.F.R. Part 111, “Current Good Manufacturing Practice in Manufacturing, Packaging, Labeling, or Holding Operations for Dietary Supplements.”

While both Parts 111 and 117 have similarities, perhaps the most striking difference is the manner in which “hazards” are approached. Under Part 117, governing foods, the last responsible party, just prior to placing a consumer product into commerce, must ensure that all hazards associated with both the manufacturing processes and ingredients in the product of commerce have been identified and properly addressed somewhere along the path of the products and ingredients being processed or manufactured. Under Part 111, governing dietary supplements, the manufacturer or distributor of the dietary supplement (product of commerce) must also identify all hazards associated with all of the ingredients and the manufacturing process but, in contrast to the requirements of Part 117, must ensure that all such hazards are addressed over the course of manufacturing the dietary supplement itself.

- Labeling: The label requirements for food and dietary supplements share many similarities. The goal of labeling requirements of both categories is to ensure consumers have all the information they require to make an informed purchasing decision. Two significant labeling issues between food and supplements include the type of nutrition facts labeling that each type of product requires and the type of structure/function claims that can be made on the different product types. The label of a food is required to bear a Nutrition Facts Panel (NFP) while a dietary supplement label requires a Supplement Facts Panel (SFP). Although the NFP and SFP are similar, they differ in what information either must or cannot be included. They have different formats, and the NFP is allowed to include the specific amounts of the dietary ingredients the product contains. Similarly, the SFP can include specific amounts of ingredients; however, the use of a proprietary blend, without denoting exact amounts of ingredients, is allowed.

The label of either a dietary supplement or a food product may contain one of three types of claims: a health claim, a nutrient content claim, or a structure/function claim. Health claims describe a relationship between a food, food component, or dietary supplement ingredient and the reduction of risk of a disease or health-related condition. Nutrient content claims describe the relative amount of a nutrient or dietary substance in a product. A structure/function claim is a statement describing how a product may affect the organs or systems of the body, but it cannot mention any specific disease. No FDA approval is required for structure/function claims; however, the manufacturer must provide the FDA with the text of the claim within 30 days of putting the product on the market. Conventional foods have no such burden. Supplement product labels containing such claims must also include a disclaimer that reads, “This statement has not been evaluated by the FDA. This product is not intended to diagnose, treat, cure, or prevent any disease.” Food labels are allowed to include structure/function claims relating to the nutritional value derived from the food’s ingredients. Dietary supplement labels are allowed to include a broader array of structure/function claims about how the product, or its ingredients, help maintain or support a healthy structure or function of the body.

***In summary, contrary to some media claims, dietary supplements are regulated on a higher level than conventional foods regarding safety, manufacturing, and labeling.***

### 1.3. What is the role of the FTC? Do both the FTC and FDA regulate the dietary supplement industry?

Dietary supplement marketers will often make statements, i.e., “claims,” about the qualities or benefits of their products. The claims made on the label or in the labeling of a dietary supplement, including some online and print marketing materials, are jointly regulated by the FDA and the Federal Trade Commission (FTC).

Supplement marketers are limited to certain types of claims, such as nutrient content claims and structure-function claims. Nutrient content claims expressly or by implication characterize the level of a nutrient in a dietary supplement, 21 C.F.R. § 101.13. Structure-function claims are statements that either 1) claim a benefit related to a classical nutrient deficiency disease and disclose the prevalence of such a disease in the U.S., 2) describe the role or characterize the mechanism by which a nutrient or dietary ingredient is intended to affect or maintain a healthy structure or function of the body, or 3) a statement the describes the general well-being from consumption of a nutrient or dietary ingredient, 21 U.S.C. §343(r)(6). Claims to diagnose, cure, mitigate, treat, or prevent a disease or injury are not permitted, as such claims can only be made on drug products, 21 U.S.C. § 321(g)(1). For example, a claim to help alleviate occasional gas or bloating is a permissible structure-function claim because occasional gas or bloating is a “healthy” structure or function of the body. A claim, however, to treat or prevent chronic gas or bloating is considered a disease claim and cannot be made about a dietary supplement. The FDA can take enforcement action in response to products deemed to include impermissible claims on their label or in their labeling. Under section 403(a) of the Act, 21 U.S.C. § 343(a), a product is misbranded if any of the information on its label or in its labeling is “false or misleading in any particular.” Also, specifically under section 403(r)(6), 21 U.S.C. § 343(r)(6), a party responsible for making a structure-function claim is required to have substantiation that such a claim is truthful and not misleading.

The role of the FTC, based on its authority under the Federal Trade Commission Act (FTC Act), 15 U.S.C. § 41 et seq, is to enforce laws outlawing “unfair or deceptive acts or practices.” The FTC’s goal is to ensure that consumers are provided with truthful and accurate information about dietary supplements so that they can make informed purchasing decisions. As a result of this, the FTC, similar to the FDA, requires that parties responsible for making claims about their products have sufficient substantiation supporting those claims.

Although they share jurisdiction, the FDA and FTC have agreed, via a memorandum of understanding, “Memorandum of Understanding Between the Federal Trade Commission and the Food and Drug Administration, that (with the exception of prescription drugs) FDA has primary jurisdiction for regulating the label and labeling of dietary supplements, while FTC has primary jurisdiction for regulating the advertising of dietary supplements” [7].

Technically, the FDA has broad authority to regulate and enforce against dietary supplements that make false or misleading claims on the product’s label or labeling. However, the FDA reserves enforcement actions relative to misbranding for technical situations (i.e. violations that are procedural, administrative, or relate to the specific requirements of labeling and packaging, rather than claims that directly threaten consumer safety) and prefers to enforce and regulate more often based on a product being deemed adulterated.

The FTC enforcement authority concerning the false advertisement of products labeled as dietary supplements is broad. For example, the FTC Act requires advertisers to avoid making false claims about their products and to have sufficient support to substantiate the claims before making them. See: In Re Pfizer Inc., 81 F.T.C. 23, 29 (1972). FTC’s authority to investigate false advertising includes the issuance of either subpoenas or Civil Investigation Demands, 15 U.S.C. §§ 9, 57b-1. A subpoena can require a responsible party to give testimony and provide production of all documents relating to an investigation, 15 U.S.C. § 49. More than likely, however, an FTC investigation into any unfair or deceptive acts or practices concerning the advertising of a dietary supplement would begin with the issuance of what is known as a Civil Investigation Demand (CID). In addition to obtaining existing documents and testimony, similar to a subpoena, a CID may also be used to require its recipient to “file written reports or answer questions,” 15 U.S.C. § 57b-1(c)(1).

***In summary, the shared jurisdiction and authority of the FDA and the FTC help ensure that marketers’ claims about the benefits and safety of dietary supplement products are truthful, not misleading, and supported by science.***

### 1.4. Besides federal regulations for dietary supplements, do state laws also regulate the category?

Yes, as a category of food, dietary supplements are regulated under federal laws and regulations as well as under any applicable state statutes, codes, or regulations. Most states, prior to the enactment of the Act (or its predecessor, the Pure Food and Drugs Act, 21 U.S.C. § 11 (1906), repealed by 21 U.S.C. § 392(a), Federal Food, Drug, and Cosmetic Act, Pub. L. No. 75–717, 52 Stat, 1040 (1938)), were regulating food safety within their own borders. Although there are clear cases where the Act clearly preempts any state laws and regulations (for example, minimum food labeling requirements), there remains today some significant activity on the part of the states to regulate certain aspects of food safety. The precise manner in which any single state regulates food, however, is dictated by the specific requirements of that state’s laws and regulations – there is no “one size fits all” set of state-level requirements.

One example of a state law impacting the supplement industry is California’s “Safe Drinking Water and Toxic Enforcement Act of 1986” – the ballot initiative known as Proposition 65 (“Prop 65”). It was designed to protect Californians from toxic chemicals, particularly in the water supply. Prop 65 directs California’s State Attorney General to keep a list of chemicals “known to the State of California to cause cancer, reproductive or developmental harm” and requires public warnings about certain exposures to these chemicals. The list of Prop 65 chemicals is now over 800, including pesticides and heavy metals, and the warnings about exposure are everywhere in California. Under Prop 65, a seller must warn about lead if it is present in amounts in excess of the “safe harbor level” of 0.5 micrograms per day – a threshold as much as 20 times lower than federal/international limits. Supplement companies selling their products in California must comply with the law regardless of where the company is based.

State consumer protection laws can also be used to address supplement products that mislead consumers. There are countless cases of plaintiff class action lawyers suing supplement marketers, big and small, in state courts for labeling discrepancies or unfounded claims. For example, plaintiffs have sued over the stated calorie content in pre-workout products containing branched-chain amino acids [8]. The potential exposure to class action lawsuits is another factor that incentivizes supplement marketers to ensure that their claims are substantiated and not misleading.

Recently, some states have taken steps, perhaps beyond their authority to do so, to enhance their regulation of dietary supplements labeled for weight loss or muscle building. For example, on 25 October 2023, New York enacted GBL 391-oo, which bans the sale of over-the-counter diet pills and dietary supplements intended for weight loss and muscle building to individuals under the age of 18. Supplements impacted by this law include those “labeled, marketed or otherwise represented for the purpose of achieving weight loss or muscle building,” and the law requires retailers, both physical stores and online vendors, to verify the age of individuals prior to sale or at the point of delivery. Other states are following suit by introducing proposed legislation. For example, in the state of Massachusetts, lawmakers have introduced Bill HD 716 to ban the sale of weight loss or muscle-building dietary supplements to any person under 18 years of age. However, unlike New York, Massachusetts does not exclude protein bars, drinks, or powders from the ban. The appropriateness of these state protectionist laws in a federally regulated market is subject to question, but they serve as an additional example of the expansive tapestry of laws and regulations overseeing the supplement market.

***In summary, while federal laws provide a baseline for the regulation of the supplement market, individual states have the authority to enact and implement their regulations.***

### 1.5. If a supplement company funds a study, does that automatically call into question the results?

The vast majority of experimental research requires funding and/or external support. While many avenues of support exist, an emerging trend over the past few decades involves industry-funded research. Industry-funded research significantly contributes to the progression of scientific research, especially in large-population and long-duration studies. However, industry-funded research is often criticized and sometimes questioned due to the potential risk of bias in research reporting and/or perceived conflict of interest involving the relationship between the research team and study sponsor.

It is reasonable to assume that all research (independent of funding sources) has the potential for bias. Concerns related to the experimental validity of industry-funded research typically pertain to perceived conflicts of interest, lack of transparency in methodology, and subjective reporting of results. There is some evidence that industry-funded research reports more favorable results compared to non-industry-funded research [9,10]. However, others report an opposite relationship [11,12]. It is important that the processes involved in securing funding for research are disclosed [13]. Research agreements should include clear details that support and protect the integrity of the researchers, research design, and academic freedom. Further, agreements should ensure that researchers are not restricted beyond a reasonable standard in capacities related to research design, timeline, or content of the publication [14].

Moreover, it has been posited that clinical trial registration is an important step toward better transparency in research [15]. By registering trials, researchers help minimize publication bias and selective reporting, while potentially enhancing the credibility and accessibility of study results [16]. A review of clinical trials submitted to *The BMJ* over four years found that improper registration, either retrospective or missing altogether, remained common, particularly among government- or foundation-funded studies [17]. Of 123 papers identified, 89.4% were retrospectively registered, and 7.3% were unregistered [17].

Industry-funded research can be reliable and accurate. Researchers can overcome perceived conflicts of interest by ensuring strong research design and methodological procedures, incorporating strategies such as independent peer review and adherence to ethical standards, and fully disclosing any relationship with the funder. To emphasize scientific rigor, transparency in components of the experimental design, including funding source, procedures for dissemination of results (i.e. ensuring research is publishable independent of the results), and indicating any conflicts or relationships, are necessary components to reduce or eliminate any potential bias.

***In summary, research agreements should include clear details that support and protect the integrity of the researchers, research design, and academic freedom. Additionally, it should be emphasized that investigators disclose any potential conflicts of interest. If an investigator does not disclose potential conflicts, it may shed a negative light on any findings related to an investigation. In addition, strong research design, studies with an appropriate sample size, as well as replicability, should be pursued with all investigations.***

### 1.6. Can diet alone provide everything you need without using supplements?

*Micronutritional adequacy: possible, but elusive* - Multiple lines of evidence show that micronutrient shortfalls are pervasive across a wide range of populations. For example, Misner [18] analyzed the dietary intakes of 70 different individuals and chose the top 20 diets with the greatest variety/number of different food types. This final sample consisted of 14 competitive athletes and 6 sedentary non-athletes. Half of the diets were calorie-excessive, and half were calorie-deficient. Men failed to meet the Recommended Daily Allowance (RDA) for 45.8% of the 10 vitamins and 7 minerals analyzed. Women failed to meet the RDA for 35.2% of these same nutrients. Collectively, micronutrient deficiencies were found in 138 of the 340 (40.5%) micronutrients analyzed. Caloric restriction, or restriction of specific foods and/or food groups, increases the likelihood of inadequate nutrient intakes. In this vein, Calton [19] analyzed the micronutrient sufficiency of four popular diet programs (Atkins, Best Life, DASH, South Beach) and found that the Reference Daily Intake (RDI) deficiency for 27 essential micronutrients ranged from 22.22 to 55.56%. RDIs, now more commonly referred to as Dietary Reference Intakes (DRIs), are guidelines that outline recommended levels of nutrient intake [20]. These reference values help estimate the amount of nutrients most people in a given population need and thus serve as useful tools for evaluating and planning healthy diets for both individuals and groups. None of the four diet programs achieved 100% of all the nutrients tested. Athletic populations tend to create their own unique set of challenges. Bodybuilders exemplify this issue, given their distinct dietary patterns characterized by both food exclusions as well as preferential overconsumption. Kleiner et al. [21] examined the pre-contest dietary habits of junior national and national-level competitors. Despite consuming adequate total calories, women were “remarkably deficient” in calcium intake. This finding is consistent with the exclusion of dairy products commonly observed in pre-competition dietary regimens. Subsequent work by Kleiner et al. [22] on nationally ranked elite bodybuilders found that men consumed 46% of the RDA for vitamin D. In contrast, women consumed 0% of the RDA for vitamin D and only 52% of the RDA for calcium. Moreover, women fell short of the recommended intakes of zinc, copper, and chromium. Serum magnesium levels in women were low despite dietary magnesium intakes above the RDA.

*Fortification, enrichment, nutrient density* – The fortification of foods by adding nutrients, and the enrichment of foods by replacing nutrients lost during processing, have been important factors in making nutrient deficiency diseases rare among the general population in developed countries like the United States [23]. However, fortification and enrichment mainly apply to grain products. Merely increasing the intake of fortified and/or enriched flour foods would be a questionable primary strategy in the quest for fulfilling micronutrient needs. Consequently, enhancing the nutrient density of the overall diet through optimized dietary composition becomes a critical step. The Mediterranean dietary pattern (characterized by its inclusion of the full range of food groups, an abundance of plant foods, as well as nuts, olive oil, and moderate amounts of wine) has a consistent track record for favorable health outcomes [24]. The Japanese dietary pattern, also known for its favorable health effects, shares commonalities with the Mediterranean model (both are rich in vegetables, beans, and fish), but the Japanese model is uniquely rich in fermented foods and seaweed. Santa et al. [25] consequently recommended a hybrid approach that integrates both dietary models to leverage their complementary benefits. It should be noted, however, that the Japanese dietary pattern may be inadequate. Shinozaki et al. [26] showed that “Japanese children and adults could improve their nutrient intake by increasing calcium, iron, dietary fiber, and potassium and reducing total and saturated fats and sodium.” Furthermore, Takiguchi et al. [27] suggested that there may be insufficient consumption of beans, green/yellow vegetables, fresh fruits, meat, and fish in older adult Japanese women. Ortega et al. [28] discovered that the Spanish population may have inadequate intake of vitamins A, D, E, and folic acid. Thus, despite the Japanese and Mediterranean diets being characterized as among the more ideal diets, individuals on those diets may still suffer from inadequate intake of various nutrients.

*Supplementation to solve potential shortfalls* - Although a food-centered approach should be taken to achieve complete nutrition, micronutrient shortcomings are likely. Limited food preferences and food intolerances among the general public can result in suboptimal ranges of healthy food selection. This problem can be exacerbated by geographic and socioeconomic limitations of food availability and/or purposeful restriction of intake among individuals pursuing weight loss. A straightforward solution is the supplementation of the nutrients deficient in the diet alone. Individual micronutrient supplementation would depend on the nature of the dietary restrictions. A systematic review by Bakaloudi et al. [29] found that veganism lacks vitamins B12, B3, B2, D, calcium, iodine, potassium, selenium, and zinc. In addition, vegan diets tend to lack omega-3 fatty acids, particularly the marine-derived EPA and DHA, thus raising the benefit of microalgal oil supplementation [30]. Moreover, O’Hearn reported that strict carnivorism tend to lack vitamin C and calcium [31]. Regarding carbohydrate-restricted diets (including Atkins-style and Paleo-style diets), a systematic review by Churuanksuk et al. [32] reported a lack of vitamin B1, B9, C, calcium, magnesium, iodine, and iron. Regardless of where the diet lies on the food restriction spectrum, the “shotgun” approach of taking a multivitamin-mineral (MVM) supplement is a simpler alternative to trying to fill individual nutrient gaps. Although the therapeutic or disease-preventive benefit of MVM use is a topic of ongoing debate [33], moderately dosed, broad-spectrum MVM supplementation is safe in the long term [34]. The potential benefits of MVM appear to outweigh any risks, which are typically negligible [35]. A growing body of evidence shows that MVM supplementation may be particularly beneficial for older populations, showing a lower prevalence of nutrient inadequacies, as well as improved immunity and cognitive function [35–39].

Of all available ergogenic compounds derived from the diet, creatine is perhaps the most prolifically studied, with a robust safety profile [40]. A burgeoning area of research is creatine supplementation for clinical/therapeutic purposes, including injury recovery, glucose control, immunity, and neurological health [41]. A maintenance dose of 3–5 g/day (more is justified in larger individuals) can saturate skeletal muscle creatine stores in approximately four weeks, while a loading scheme of 20–25 g/day can accomplish this in 5–7 days. Regardless of whether a loading phase is employed, attaining ergogenic levels of creatine without supplementation is impractical for most people. Typical mixed, omnivorous diets provide 1–2 g creatine per day. Rich dietary sources of creatine are beef, fish, pork, and poultry. To attain an ergogenic maintenance dose of creatine (3–5 g), one would need to consume approximately 1–2 kg (2.2–4.4 lb.) of meat per day. It should also be noted that plants are devoid of creatine. The challenges posed by impractical meat consumption volumes and the absence of creatine in plant-based diets can be effectively addressed through dietary creatine supplementation. It should nonetheless be stated that one does not need creatine to “survive.” Distinctions should be made between “optimization” versus “necessity.”

***In summary, even the most notable diets that are posited as the healthiest (i.e. Japanese and Mediterranean) may not meet the recommended levels of various nutrients. If one’s goals include optimizing health and performance, dietary supplements appear to be helpful.***

### 1.7. Is it necessary to conduct randomized controlled trials (RCTs) on dietary supplements?

The necessity of conducting RCTs on dietary supplements is a complex issue that depends on various factors, including regulatory requirements, the nature of claims made, and the scientific evidence available. It’s important to note that the requirements can differ significantly between regulatory frameworks, such as those in the United States and Europe [42].

In the United States, contrary to common misconception, there is no specific legal requirement for RCTs to substantiate claims for dietary supplements. The Food and Drug Administration (FDA) does not mandate RCTs for dietary supplements. Instead, the FDA looks at the totality of scientific evidence to substantiate claims [43]. Their approach is flexible and nonspecific, allowing for various types of evidence to support claims. The Federal Trade Commission (FTC), which regulates advertising claims, has published guidance suggesting a preference for RCTs as “competent and reliable scientific evidence” [44]. However, it’s crucial to understand that this is guidance, not law. The FTC’s stance on RCTs has been challenged by industry stakeholders, and a significant Federal ruling rejected the FTC’s requirement for two RCTs to substantiate claims [45,46].

Both the FDA and FTC allow for flexibility in the type of evidence used to substantiate claims [43,44]. The focus is on the overall quality and relevance of the evidence rather than adhering to a strict requirement for RCTs. This means that other forms of scientific evidence, such as epidemiological studies, in vitro studies, and animal studies, can contribute to the substantiation of claims when considered as part of the totality of evidence.

Under the Dietary Supplement Health and Education Act of 1994 (DSHEA), manufacturers can make “structure and function claims” describing how a nutrient or dietary ingredient affects normal body structures or functions [47]. These claims do not require FDA pre-approval but must be truthful, not misleading, and include a disclaimer stating the FDA has not evaluated the claim. For more specific health claims, particularly those related to the treatment or prevention of disease, a higher standard of evidence is typically expected, though not necessarily in the form of RCTs [43,44]. In the European Union, the approach is somewhat different. Any ingredient not consumed significantly before May 1997 is considered a “novel food” and requires extensive safety data, which may include clinical trials [42]. The European Food Safety Authority (EFSA) generally requires robust scientific evidence to substantiate health claims, often preferring RCTs, but this is not an absolute requirement [42].

While RCTs are often considered the gold standard of evidence, they serve several important purposes beyond regulatory compliance. They provide scientific validation by establishing causality, enhancing consumer confidence, assisting in quality assurance, and can help substantiate specific health claims [43,44,48]. However, the necessity of conducting RCTs should be evaluated on a case-by-case basis, considering the specific claims being made, the existing body of evidence, and the feasibility of conducting such trials. Manufacturers have significant responsibilities regardless of whether they conduct RCTs. Ensuring product safety, adhering to Good Manufacturing Practices (GMP) [49], and ensuring that all claims are truthful and not misleading is paramount.

***In summary, while RCTs are valuable tools in substantiating health claims for dietary supplements, they are not a universal legal requirement, particularly in the United States. The regulatory landscape allows for a more flexible approach, considering the totality of evidence rather than mandating a specific type of study. As the industry evolves, there’s a trend towards increased scientific substantiation (i.e. product-specific substantiation). However, the current state of regulation emphasizes the importance of a comprehensive, evidence-based approach to substantiating claims, which may or may not include RCTs depending on the specific circumstances.***

### 1.8. How safe are supplements compared to OTC drugs?

The Food and Drug Administration (FDA) defines a drug as “a substance intended for use in the diagnosis, cure, mitigation, treatment, or prevention of disease.” [50] Drugs can be further categorized as those requiring a prescription from a licensed healthcare provider (Legend Drugs) and those deemed safe for self-administration to treat self-diagnosed issues without the need of a healthcare professional (OTC drugs) [51]. The history of drug regulation initially started with little oversight, producing a landscape of uncertainty regarding the effectiveness and safety for the consumer. It wasn’t until the Federal Food, Drug, and Cosmetic Act of 1938 that transparency regarding the content, dosage, and verification of medication claims became a required standard. Since then, numerous amendments and acts have been signed into law [51].

*OTC Drug Use & Safety -* A crucial element that cannot be overlooked in the utilization of OTC drugs is the reliance on individuals without formal medical training to correctly correlate a symptom with a diagnosis and select the proper drug for management. A review of the most common selling OTC drugs indicates that most symptoms being managed involve pain, cough/congestion & flu, allergies, constipation/diarrhea, and heartburn [52,53]. Acetaminophen (aka paracetamol or N-acetyl-p-aminophenol) is one of the most commonly used antipyretic/analgesic medications worldwide, with use spanning a wide range of ages from pediatrics to geriatric care [52]. While its use has been generally considered safe, it is not without its significant side effects. Potential for drug-induced hepatotoxicity leading to hepatocellular necrosis and acute liver failure when taken in doses exceeding the manufacturer’s recommendations is a well-known concern. Data from 2017 indicated that acetaminophen toxicity was responsible for approximately 500 deaths, as well as 50,000 emergency room visits and 10,000 hospitalizations, [54]. Several variables have been identified that impact the potential for adverse reactions. Therapeutic dosages can vary significantly based on the patient’s age and co-morbidities. Etiologies leading to derangement in hepatic function, such as metabolic dysfunction-associated steatotic liver disease (MASLD), metabolic-associated steatohepatitis (MASH), and cirrhosis, require a significant reduction in the maximum total daily dose of acetaminophen. The mechanism of this is theorized to be in part due to derangement in the activity of cytochromes CYP3A4 and CYP2E1, two enzymes that are highly associated with the production of the metabolic byproducts, which can cause significant hepatocellular damage when in excess [55]. For this population, a maximum daily dose of 2 grams per day is recommended, which is half the recommended maximum dose for the average population. What makes this particularly alarming is the increased prevalence of MASLD in the United States and globally. It is estimated that approximately 30% of the global adult population has MASLD, and it has become the most common form of liver disease in pediatric populations [56]. Pediatric dosing is based on weight with a maximum recommended dose of 60–75 mg/kg/day. Currently, the impact of obesity in the pediatric population and the resulting increased volume of medication administered is not fully known. However, what is known is that children with obesity are impacted with the same systemic inflammation as adults and may have functional derangement of hepatic cells, opening them up to potential drug-induced hepatotoxicity even at dosages originally deemed safe [57]. With use stemming back as far as the 1940s, H1 antihistamines continue to be a core treatment for allergic conditions such as allergic rhinitis, rhinosinusitis, urticaria, and other related symptoms [58]. Acting as inverse agonists to the 7-transmembrane G protein-coupled H1 receptor, the effectiveness and potential side effect profile are largely based on generation class. First-generation antihistamines readily cross the blood-brain-barrier (BBB), leading to inverse agonism at CNS receptors. This inhibition of neurotransmission in histaminergic neurons results in many of the concerning side effects of impaired alertness, alteration in cognition, and, in severe cases, somnolence [59]. Additionally, the poor ability of first-generation antihistamines to target specific H1 receptors increases the risk of adverse effects due to unintentional interactions with other receptor types. These undesired effects include but are not limited to postural hypotension due to alpha-adrenergic receptor antagonism, QT prolongation, and increased rate of ventricular arrhythmias due to cardiac ion channel interaction, and others [60]. It is important to note that antihistamines represent an area of OTC medication where continued advancements in research have produced an improvement in both effectiveness and side effect mitigation. The second-generation antihistamines lower lipid solubility and variation in ion charge significantly impair crossing of the BBB, thereby attenuating many of the most concerning side effects [61].

*Dietary Supplement Use & Safety -* Data investigating the use of dietary supplements in 2017 through 2018 by the National Center for Health Statistics found that 57.6% of adults 20 years of age and older reported using dietary supplements. This percentage increased with age, with use increasing to 80.2% for those over the age of 60. The top three most common types of dietary supplements used were multivitamin-minerals, vitamin D, and omega-3 fatty acids [62]. A review of three prospective cohort studies totaling 390,124 participants investigating the impact to mortality of multi-vitamins on mortality in generally healthy adults failed to identify significant adverse events [63]. Investigation into the extent to which dietary supplement consumption has resulted in the need for emergency care appears to be greatest in young adults experiencing subjective cardiac symptoms (palpitations, angina, tachycardia) resulting from weight loss products and high caffeine-containing pills/beverages. The majority of these incidents (89.9%) resulted in discharge from the ER without hospitalization [64]. Systematic reviews of case reports citing dietary sport supplements as suspected causative agents of adverse events have noted that many of these encounters relied on clinical reasoning to formulate conclusions instead of a structured causality assessment method [65].

Today, the use of OTC medication represents a substantial portion of medical management in the general population. Laws and amendments spanning over 86 years have resulted in an improvement in safety and transparency. However, with well over 100,000 different OTC drugs available in the United States, severe adverse effects stemming from improper dosage, drug-to-drug interaction, and varying effects on different age populations are still of great concern. Advancements in the research of OTC medication continue to be attempted, working to decrease side effects via numerous methods such as increasing drug-receptor specific interaction. Consumers need to be mindful of the real potential negative impacts associated with these drugs and not assume them benign simply due to their lack of need for a physician’s prescription for use.

***In summary, the landscape of OTC drugs has evolved considerably from their early introduction as a collection of substances without a clear description of use, ingredients, or potential side effects. However, with well over 100,000 different OTC drugs available in the United States, severe adverse effects stemming from improper dosage, drug-to-drug interaction, and varying effects on different age populations are still of great concern. It is recommended that individuals seek advice from trained professionals and provide detailed accounts of all medications (prescribed & OTC).***

### 1.9. Where can consumers find accurate information about supplements?

Consumers, healthcare professionals, and scientists need reliable, evidence-based resources in the crowded dietary supplement market to navigate an overwhelming array of claims. Accurate information is critical for ensuring safety, optimizing effectiveness, and avoiding the pitfalls of misinformation. This section highlights key sources for finding trustworthy supplement information, including independent certifications, professional organizations, consumer resources, and strategies to combat misinformation.

#### 1.9.1. Independent testing and certification programs

Third-party certification programs are essential for verifying the quality, safety, and labeling accuracy of dietary supplements. For consumers, particularly athletes and other high-risk populations, these certifications serve as a vital checkpoint in the supplement selection process.

- Informed-Choice/Informed-Sport: These globally recognized certification programs test supplements for prohibited substances, contaminants, and labeling accuracy. Informed-Choice focuses on general consumers, while Informed-Sport caters to elite athletes subject to anti-doping regulations. Both programs are trusted worldwide for their rigorous quality assurance standards [66]. Informed Choice, for instance, will have pre-certification batch testing of at least three samples from three different production batches [67]. Informed Sport tests every single batch of a certified product (i.e. only finished products are tested) [68].
- NSF Certified for Sport®: Widely trusted by professional athletes and organizations, NSF tests supplements to ensure they are free from banned substances, contaminants, and unsafe levels of heavy metals. Products certified by NSF also undergo stringent quality control audits [69]. In addition to initial batch testing, NSF also performs ongoing monitoring, which includes annual facility audits and periodic marketplace sampling where products are anonymously purchased and tested to ensure continued compliance [70].
- Banned Substances Control Group (BSCG): Known as “the gold standard in banned substance certification,” BSCG ensures that supplements meet stringent safety and quality criteria, offering peace of mind for athletes and consumers [71]. The Banned Substances Control Group (BSCG) performs batch testing by analyzing each finished product lot for banned substances prior to certification. Every production lot undergoes individual testing against a comprehensive panel of over 500 compounds, including substances prohibited in sport and other pharmaceutical contaminants [72].
- U.S. Pharmacopoeia (USP): Supplements that carry the USP Verified Mark have been tested for purity, potency, and quality, ensuring that the ingredients listed on the label match what is in the product [73].

#### 1.9.2. Government resources: NIH Office of Dietary Supplements (ODS)

The National Institutes of Health’s Office of Dietary Supplements (ODS) provides accessible, evidence-based resources for healthcare providers and the public. Its fact sheets offer detailed information on supplement safety, efficacy, dosage, and potential interactions. The ODS also hosts the Dietary Supplement Label Database, a searchable tool that includes information on thousands of supplements, helping consumers and professionals make more informed choices [74].

#### 1.9.3. Military-Specific guidance: Operation Supplement Safety (OPSS)

The Department of Defense’s Operation Supplement Safety (OPSS) initiative is an invaluable resource for military personnel and their families. OPSS provides tools such as the “High-Risk Supplement List” and educational resources to mitigate risks associated with dietary supplements. The program offers a free supplement evaluation tool that helps service members assess product safety and compliance with military regulations. OPSS also delivers tailored resources for healthcare providers, leaders, and civilians in the military community, addressing the unique challenges they face in a high-performance environment [75].

#### 1.9.4. The Journal of the International Society of Sports Nutrition (JISSN)

The Journal of the International Society of Sports Nutrition (JISSN) is a global authority in sports nutrition and supplementation [76]. Comprised of world-class researchers, registered dietitians, medical doctors, and legal experts, the JISSN delivers evidence-based guidance that is both practical and scientifically rigorous. One of the JISSN’s hallmark contributions is its position stands, which are peer-reviewed documents addressing critical topics such as protein intake, creatine supplementation, and ergogenic aids. These position stands are considered gold standards in sports nutrition, providing healthcare providers and consumers with clear, actionable recommendations.

#### 1.9.5. Consumer-Focused platforms

Accessible platforms designed for consumers help simplify the complexities of supplement evaluation while combating misinformation.

- Examine.com: A comprehensive and evidence-based database for consumers, Examine.com reviews and summarizes research on supplements, providing clear and accessible insights on their efficacy and safety [77].
- Labdoor: Labdoor independently tests and ranks supplements based on ingredient quality, label accuracy, and purity. Its user-friendly reports allow consumers to make informed decisions about which brands and products to trust [78].
- Truth in Advertising (TINA): TINA exposes deceptive marketing practices in the supplement industry, empowering consumers to identify and avoid misleading claims [79].

For consumers overwhelmed by conflicting information, these platforms provide clear, actionable insights rooted in evidence and transparency. On the other hand, social media platforms, such as Instagram and TikTok, have become sources of information, both good and bad, for athletes and the general population. Credentials, such as an M.D., R.D., D.O., and PhD., can function as vetting tools, but this does not ensure that accurate, high-quality information will come from those individuals.

#### 1.9.6. Combating misinformation and recognizing conflicts of interest

In today’s digital landscape, misinformation about dietary supplements spreads rapidly through social media, unregulated websites, and influencer endorsements. Claims of “miracle cures” and supplements marketed as substitutes for prescription medications are common red flags. To counteract these trends, consumers should:

1. Verify claims through trusted scientific databases/peer-reviewed journals like the JISSN.
2. Look for third-party certification insignias like Informed-Choice, NSF, BSCG, and USP.
3. Consult healthcare professionals, such as registered dietitians, for personalized advice.

However, even healthcare professionals are not immune to conflicts of interest. Financial relationships with supplement companies, particularly in today’s influencer-driven culture, can compromise objectivity. Consumers should feel empowered to inquire about potential ties between providers and supplement manufacturers to ensure unbiased guidance.

***In summary, finding accurate supplement information requires a multi-pronged approach that includes leveraging independent certifications, consulting professional organizations like the ISSN/JISSN, and utilizing consumer-focused platforms. Resources such as NIH ODS, OPSS, Examine.com, Labdoor, and TINA provide clarity in a market saturated with misinformation. By critically evaluating claims and utilizing these trusted tools, consumers, healthcare professionals, and scientists can navigate the supplement market with confidence and clarity.***

### 1.10. Why does the NIH fund dietary supplement research related to disease, yet findings cannot be marketed by supplement companies?

While some health claims have been approved for dietary supplements and foods, only products classified as approved drugs, over-the-counter medications (OTCs), or medical foods may legally be marketed to treat, cure, or prevent disease. Accordingly, dietary supplements marketed in the United States that make claims about a) nutrient content, b) health-related effects, or c) effects on bodily structure or function are required to include the standard disclaimer:

“**This product has not been evaluated by the Food and Drug Administration. This product is not intended to diagnose, treat, cure, or prevent any disease**.” [80] In addition to the claim categories available to foods (e.g. related to taste or quality), two other types of claims are permitted for dietary supplements: d) benefits related to the correction of a nutrient deficiency, provided the prevalence of that deficiency in the U.S. population is disclosed, and e) claims that refer to general well-being [81].

If a dietary supplement is advertised as treating diseases, serious health conditions, or as having effects similar to drugs, the company selling it is likely breaking a key rule under the Dietary Supplement Health and Education Act (DSHEA) of 1994. This kind of false marketing (i.e. promoting a supplement as if it were a drug) is one of the most common reasons the FDA and FTC take action against supplement companies.

The National Institutes of Health (NIH), particularly through its Office of Dietary Supplements (ODS), continues to support research on dietary supplements, including their role in health maintenance and disease prevention. The ODS was established by DSHEA, and its mandate includes the scientific investigation of the potential health benefits of supplements. DSHEA outlines two primary objectives for the ODS:

“(1) to explore more fully the potential role of dietary supplements as a significant part of the efforts of the United States to improve health care and,

(2) to promote scientific study of the benefits of dietary supplements in maintaining health and preventing chronic disease and other health-related conditions.

The ODS Director is charged with coordinating NIH research related to dietary supplements, particularly studies evaluating their role in mitigating the risk of diseases such as heart disease, cancer, osteoporosis, cataracts, and others. Additionally, the Director also provides guidance to other federal health agencies regarding supplement-related research and regulatory considerations [82]. Therefore, from its inception, the ODS’ mandated research objective has been to investigate dietary supplements with the intention of determining whether specific Authorized or Qualified Health Claims could be applied to the safe use of a given dietary supplement ingredient. For example, a dietary supplement that provides adequate doses of Calcium and Vitamin D could be marketed using the Authorized Health Claim, “Adequate calcium and vitamin D as part of a healthful diet, along with physical activity, may reduce the risk of osteoporosis in later life.”

***In summary, it is not legally accurate to contend that dietary supplements are categorically prohibited from being marketed with claims pertaining to disease prevention or health conditions studied under NIH auspices. Claims derived from NIH Office of Dietary Supplements-funded research may be eligible for marketing use, provided they are supported by sufficient scientific evidence and undergo the appropriate regulatory review and authorization processes.***

### 1.11. What is the size of the dietary supplement industry compared to the pharmaceutical industry?

The dietary supplement industry in the United States for the year 2023 was approximately a $61.1 billion dollar category [83]. Some of the companies that generate larger revenue in the dietary supplement space also retail or sell items that are not dietary supplements. For example, in 2022, Amway globally generated $8.1 billion in sales, and Herbalife generated $5.1 billion [84]. As many individuals purchase a variety of products through Amazon, it is noteworthy to realize that Amazon sold $12.6 billion in vitamins, minerals, and related supplements in 2023. The following companies appear to be the top-selling companies on Amazon in the dietary supplement space: Garden of Life, Optimum Nutrition, NOW Foods, Vital Proteins, Pure Encapsulations, Nature Made, Nutricost, Liquid I.V., MaryRuth Organics, and Nature’s Bounty [85]. For many years, GNC has been considered the market leader in selling and retailing dietary ingredients and supplements. GNC’s imprint on the market has changed, resulting in 2023 sales of $2.3 billion (~72% less than Amazon). Recent business data has 2023 revenue in the US for the dietary supplements industry at ~$54 billion. The US pharmaceutical business market is ~$670 billion, whereas, for dietary supplements, it is ~$61 billion. Thus, the US pharmaceutical market is more than 12x the size of the US supplement market. For perspective, while it is a few years back, in 2009, Pfizer (a pharmaceutical company) purchased another pharmaceutical company (Wyeth) for $68 billion. The perspective to keep is that Pfizer’s purchase was at a sale price larger than that of the entire dietary supplement industry [86].

***In summary, the US pharmaceutical industry is more than twelve times the size of the supplement market.***

### 1.12. How can I know if a dietary supplement is safe and free of banned substances?

Studies have found that dietary supplements may contain contaminants. Panneerselvan et al. found the presence of microplastics in dietary fiber supplements [87]. A study from Food Science & Nutrition examined 41 dietary supplements from terrestrial plants or microalgae, and found that 68% of the samples were contaminated with heavy metals [88].

Dietary supplements are regulated by the Food and Drug Administration as a subset of foods. The Dietary Supplement Health and Education Act of 1994 was codified into law, making safety a paramount part of product development through the Good Manufacturing Practices aspect of the law. The Code of Federal Regulations (21 CFR 117) helps ensure the relative manufacturing safety of food and dietary ingredients by applications of hazard analysis and risk-based preventive controls for human food and supplements as standards. The Code of Federal Regulations (21 CFR 111) also mandates various stringent controls and testing to help ensure that the dietary ingredient is safe for human consumption. Being that the GMPs are mandated, there is a level of presumed safety, too, with foods and dietary supplements. Food and dietary supplement companies are also regulated under the Food Safety Modernization Act (FSMA), yet another step to help ensure safety in our nutrition supply [89]. In brief, FSMA, signed into law in 2011, shifted the food safety focus from being reactive (i.e. responding to contamination) to being preventive or proactive (i.e. preventing contamination prior to its occurrence) [90].

If a dietary ingredient was not on the US market before 15 October 1994, then to bring it to the market, companies need to go through either a Generally Recognized as Safe evaluation (GRAS) or be positioned to be submitted to the FDA for review as a New Dietary Ingredient (NDI). The use of GRAS evaluations has a long history within food safety and the FDA [91]. It is one method of determining the safety and tolerability of an ingredient. Companies may also submit an NDIN to the FDA for evaluation, which is another regulatory method for a safety review of the ingredient or product before it goes to market (pre-market notification).

To determine if a dietary supplement is free of banned substances (i.e. a substance banned by WADA), companies can obtain third-party testing for evaluation and demonstration that the product is what it says it is and that it has no banned substances. The World Anti-Doping Association (WADA) and the United States Anti-Doping Association have published educational materials for general knowledge regarding banned substances. USADA currently recommends that if an athlete is interested in taking a supplement, they look for the product to be NSF Certified for Sport [92]. In the past, USADA has also worked with and endorsed the Banned Substances Control Group testing and certification, along with Informed Choice® and Informed Sport®, while noting internationally that the testing by Kölner Liste® (Germany, Cologne List) or by the Australian HASTA group also is reassurance on the product being free from banned substances [93].

***In summary, organizations such as Informed Choice®, Informed Sports®, NSF, etc., offer services to determine the presence of banned substances.***

## 2. Conclusions

Based on our scientific and legal evaluation, we conclude that:

1. The primary regulatory bodies responsible for the dietary supplement industry are the FDA (as well as the FTC).
2. Dietary supplements are regulated on a higher level than conventional foods regarding safety, manufacturing, and labeling.
3. The shared jurisdiction and authority of the FDA and the FTC help ensure that marketers’ claims about the benefits and safety of dietary supplement products are truthful, not misleading, and scientifically supported.
4. Although federal laws provide a baseline for the regulation of the supplement market, individual states have the authority to enact and implement their own regulations.
5. It should be emphasized that investigators disclose any potential conflicts of interest.
6. It is evident that diet may not sufficiently meet all of one’s nutritional needs. Factors such as an individual’s health status, physical activity level, personal goals, and other lifestyle factors (e.g. dietary preferences, food accessibility, etc.) may affect one’s nutritional needs.
7. As the industry evolves, there’s a trend toward increased scientific substantiation (i.e. product-specific substantiation).
8. The number of adverse effects from drugs exceeds dietary supplements. This is due in part to the prevalence of use.
9. In summary, finding accurate supplement information requires a multi-pronged approach that includes professional organizations/journals such as the ISSN/JISSN, the NIH Office of Dietary Supplements (ODS), as well as consumer-based platforms such as Labdoor, etc.
10. The ODS undertakes a lengthy, resource-intensive process, which is funded by taxpayer dollars, before implementing even modest changes; moreover, some of its research may subsequently be leveraged by industry for marketing purposes.
11. The US pharmaceutical industry is more than twelve times the size of the dietary supplement market.
12. Organizations such as Informed Choice®, Informed Sports®, NSF, etc. offer services to determine the presence of banned substances.
